# The progress of m6A methylation-regulated non-coding RNAs in osteogenic differentiation and osteoporosis

**DOI:** 10.1016/j.ncrna.2025.11.005

**Published:** 2025-12-02

**Authors:** Yongbin Wang, Baicheng Ma, Jiashuo Qiu, Likun Yu, Jianjun Xiong, Qianfu Yu, Xingnuan Li, Haichun Liao, Youen Huang, Shan He

**Affiliations:** a University of Shanghai for Science and Technology, South Campus of Jun Gong Road, Yangpu District, 200093, Shanghai, China; b Jiangxi Provincial Key Laboratory of Cell Precision Therapy, School of Basic Medical Sciences, Jiujiang University, Jiujiang, 332005, Jiangxi, China

**Keywords:** m6A, Non-coding RNA, Osteogenic differentiation, Osteoporosis, Epigenetic modifications, Axial regulation

## Abstract

N6-methyladenosine (m6A) is the most abundant internal modification of eukaryotic RNA, and has increasingly been recognized as a critical regulator of gene expression at both the transcriptional and post-transcriptional levels. The m6A modification process is highly dynamic and reversible, governed by methyltransferase writers (e.g., METTL3, METTL14, WTAP), demethylase erasers (such as FTO and ALKBH5), and specific readers (including YTHDF and IGF2BP protein families) that interpret the modification and mediate its downstream effects. Accumulating evidence indicates that m6A-mediated regulation of non-coding RNAs (ncRNAs) plays a pivotal role in osteogenic differentiation, a process central to bone formation. Impaired osteogenesis, which contributes to decreased bone mass, is closely linked to the onset and progression of osteoporosis. This review summarizes recent progress on the interplay between m6A modification and ncRNAs in osteogenic differentiation, and outlines a regulatory framework, the m6A-ncRNA-target gene axis, that includes m6A modification, ncRNA interactions, and downstream signaling pathways, such as Wnt/β-catenin and BMP/Smad, and discusses their potential as biomarkers or therapeutic targets for osteoporosis, with the goal of providing new insights into the epigenetic mechanisms underlying osteoporosis and to identify potential molecular targets for future therapeutic strategies.

## 1 Introduction

Osteoporosis is a chronic, systemic skeletal disorder characterized by reduced bone mineral density and deterioration of bone microarchitecture, resulting in increased bone fragility and a high risk of fractures. Such fragility fractures not only compromise physical health, but also diminish overall quality of life [1]. A wide range of factors contribute to osteoporosis, with menopause and aging among the most prominent [2]. Of these, aging represents the single greatest risk factor, and the rapid demographic shift toward an older global population has led to a sharp increase in disease prevalence. Consequently, osteoporosis has become one of the most pressing global public health issues [3].

The fundamental pathological mechanism of osteoporosis is an imbalance in bone remodeling, specifically between bone formation and bone resorption [4]. Most current therapies primarily target the inhibition of bone resorption. While such treatments can effectively slow disease progression, they are often associated with adverse side effects and limited long-term benefit. This has spurred growing interest in approaches that instead promote osteoblast-driven bone formation as a more favorable therapeutic strategy [5]. A variety of cellular and molecular factors influence osteogenic differentiation, but recent attention has centered on RNA epigenetic modifications as key regulators of this process. Among these, m6A methylation, the most prevalent internal RNA modification in eukaryotes, has been the focus of considerable attention due to its broad regulatory roles [6]. Approximately one-third of mammalian mRNAs are estimated to carry m6A marks, with an average of three to five methylation sites per transcript [7]. In addition to being widely distributed, m6A modifications can broadly impact gene expression, tissue homeostasis, and developmental processes. Given these roles, dissecting how m6A contributes to osteoporosis pathogenesis, particularly through its regulation of ncRNAs during osteogenic differentiation, is crucial for deepening our understanding of epigenetic mechanisms in bone biology. Such insights hold promise for the early detection, risk assessment, and precision treatment of osteoporosis.

## 2 m6A modification: an overview

m6A methylation represents a reversible and tightly regulated form of RNA modification, maintained through the dynamic interplay of three major classes of proteins: writers, which catalyze the deposition of methyl groups; erasers, which remove these modifications; and readers, which recognize m6A sites and mediate their functional consequences. This finely tuned system plays an essential role in cellular differentiation, tissue development, and the preservation of physiological homeostasis [8].

The writer-mediated deposition of m6A is carried out by a multicomponent methyltransferase complex. Its catalytic core is composed of methyltransferase-like protein 3 (METTL3), methyltransferase-like protein 14 (METTL14), and Wilms tumor 1-associated protein (WTAP), which together ensure precise methylation of target RNAs. Several additional cofactors provide auxiliary functions, including KIAA1429 (also known as VIRMA), METTL16, RNA-binding motif proteins RBM15 and RBM15B, and zinc finger CCCH domain-containing protein 13 (ZC3H13). The complex uses S-adenosylmethionine (SAM) as the methyl donor to add a methyl group to the N6 position of adenosine residues [9]. Within this complex, METTL3 functions as the primary catalytic enzyme, directly methylating target mRNAs. METTL14 acts as a stabilizing partner, forming a heterodimer with METTL3 to enhance methylation efficiency, while WTAP ensures appropriate localization and stabilization of the enzyme complex [10,11]. KIAA1429 contributes to site-specific methylation by directing the methyltransferase complex to selected transcript regions [12], whereas RBM15/15B recruit the complex to specific RNA motifs [13]. ZC3H13 anchors the complex within the nucleus, ensuring proper m6A deposition [10]. In addition, METTL16 modifies select RNA substrates, including small nuclear RNAs (snRNAs) [14]. Together, these components orchestrate a highly coordinated system that regulates the installation of m6A modifications.

The dynamic reversibility of m6A methylation is mediated by demethylases, which remove the modification through oxidative reactions. Two such enzymes have been identified to date: fat mass and obesity-associated protein (FTO) and α-ketoglutarate-dependent dioxygenase homolog 5 (ALKBH5). Both belong to the Fe(II)/α-ketoglutarate-dependent dioxygenase family and catalyze the oxidative demethylation of adenosine residues, thereby restoring RNA to its unmethylated state [15].

**Fig. 1:**
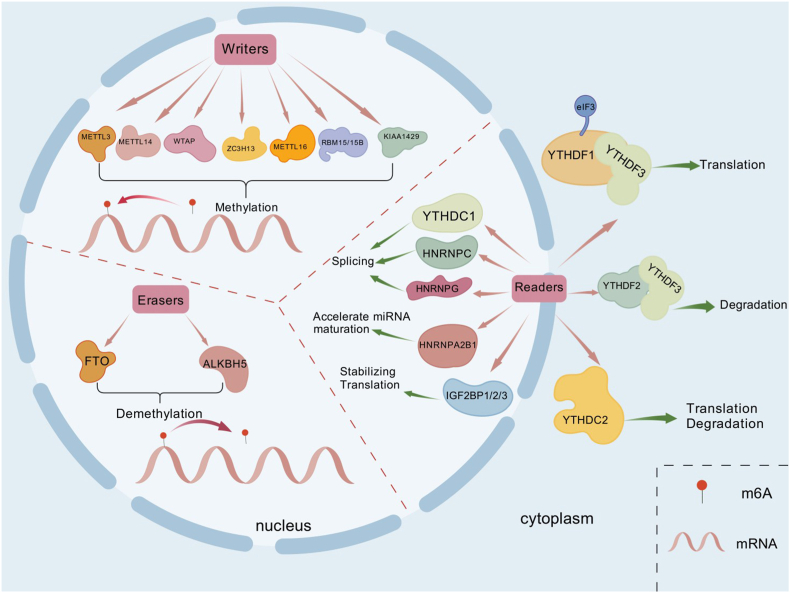
**The mechanisms governing m6A RNA methylation.** Transcript m6A methylation occurs in a dynamic, reversible manner governed by m6A methyltransferases (writers), m6A demethylases (erasers), and m6A recognition factors (readers). These complex mechanisms influence cellular differentiation, tissue development, and physiological homeostasis.

The biological functions of m6A-modified transcripts are executed by reader proteins that selectively bind to methylated sites and influence RNA metabolism, including splicing, export, translation, and degradation. Major classes of readers include the YTH domain-containing family, the insulin-like growth factor 2 mRNA-binding proteins (IGF2BP1/2/3), and heterogeneous nuclear ribonucleoproteins (HNRNPs) [16]. The YTH family can be subdivided by cellular localization, and includes nuclear-localized YTHDC1, together with cytoplasmic-localized YTHDF1/2/3 and YTHDC2 [17]. The nuclear reader YTHDC1 recognizes m6A-modified precursor RNAs and recruits splicing regulators such as serine/arginine-rich splicing factor 3 (SRSF3) to control alternative splicing and nuclear export [18]. YTHDC2 contains a helicase domain, and modulates both translation and degradation of methylated transcripts [18]. In the cytoplasm, YTHDF proteins exhibit complementary roles, with YTHDF1 enhancing translational initiation by interacting with EIF3, while YTHDF2 accelerates mRNA decay for m6A-modified transcripts [19]. YTHDF3 functions in both manners, as it supports both translation (via cooperation with YTHDF1) and degradation (through interaction with YTHDF2) [20]. HNRNP family members (HNRNPA2B1, HNRNPC, HNRNPG) function in a mechanistically distinct manner, as they do not directly recognize the methyl group, but m6A-induced alterations in RNA secondary structure expose binding motifs that enable HNRNP association and the regulation of splicing or stability [21]. For instance, HNRNPA2B1 has similar functions to METTL3, interacting with microprocessor complex proteins DGCR8 and Drosha to enable primary miRNA (pri-miRNA) processing [22]. IGF2BP1/2/3 function as reader proteins capable of recognizing m6A-modified RNAs containing the UGGAC motif in a specific manner, binding via their KH3–4 domains and recruiting HuR and other stabilizing factors. Through their actions, these proteins help to preserve transcript stability while promoting translational activity at baseline and under stress conditions (Fig. 1) [23].

## 3 The mechanistic functions of m6A-Modified non-coding RNAs in osteoporosis

### 3.1 Introduction to non-coding RNAs

Non-coding RNAs represent a broad category of RNA molecules that lack protein-coding capacity but account for nearly 98 % of the mammalian transcriptome. Historically dismissed as “junk” products of evolution, ncRNAs are now recognized as crucial regulators of gene expression and cellular homeostasis [24]. Increasing evidence demonstrates that ncRNAs participate in multiple layers of gene regulation, including transcriptional and post-transcriptional control, RNA maturation and modification, RNA stability, and protein synthesis [25]. Based on their biological roles, ncRNAs are generally divided into two groups. Housekeeping ncRNAs such as transfer RNAs (tRNAs), ribosomal RNAs (rRNAs), small nuclear RNAs (snRNAs), and small nucleolar RNAs (snoRNAs) are ubiquitously expressed, remain relatively stable across conditions, and provide essential support for basic cellular physiology. By contrast, regulatory ncRNAs, which include microRNAs (miRNAs), long non-coding RNAs (lncRNAs), small interfering RNAs (siRNAs), and Piwi-interacting RNAs (piRNAs), exert more dynamic and condition-specific control over gene expression, RNA processing, and cellular functions [26]. Circular RNAs (circRNAs), which arise from back-splicing of precursor mRNAs to form covalently closed circular transcripts, lack a 5′ cap pr 3′ poly(A), conferring them strong resistance to exonuclease-mediated degradation, making them unusually stable. CircRNAs display dual characteristics of both housekeeping and regulatory ncRNAs due to their variable lengths and ability to act in bidirectional regulatory pathways [27]. Among these known regulatory ncRNAs, miRNAs, lncRNAs, and circRNAs are currently at the forefront of research owing to their prominent roles in development, disease pathogenesis, and epigenetic regulation.

miRNAs are small (20–24 nt) regulatory RNAs that typically repress gene expression by binding complementary sequences in the 3′ untranslated regions (3′-UTRs) of target mRNAs, leading either to translational inhibition or mRNA degradation [28]. lncRNAs exceed 200 nucleotides in length, and represent a structurally and functionally diverse group implicated in transcriptional regulation, RNA splicing, chromatin modification, and translation control. They have become recognized as key elements of the epigenetic regulatory network [29]. circRNAs, usually averaging ∼500 nt in length, are distinguished by their closed-loop architecture [30], which provides strong sequence conservation and stability, making them attractive candidates for biomarker development [31]. In addition to sequestering miRNAs through a miRNA sponge-like activity [32], circRNAs can interact with RNA-binding proteins or even serve as translation templates, thereby modulating transcriptional and post-transcriptional pathways [33].

### 3.2 m6A-dependent regulation of osteogenic differentiation via miRNAs

**Fig. 2:**
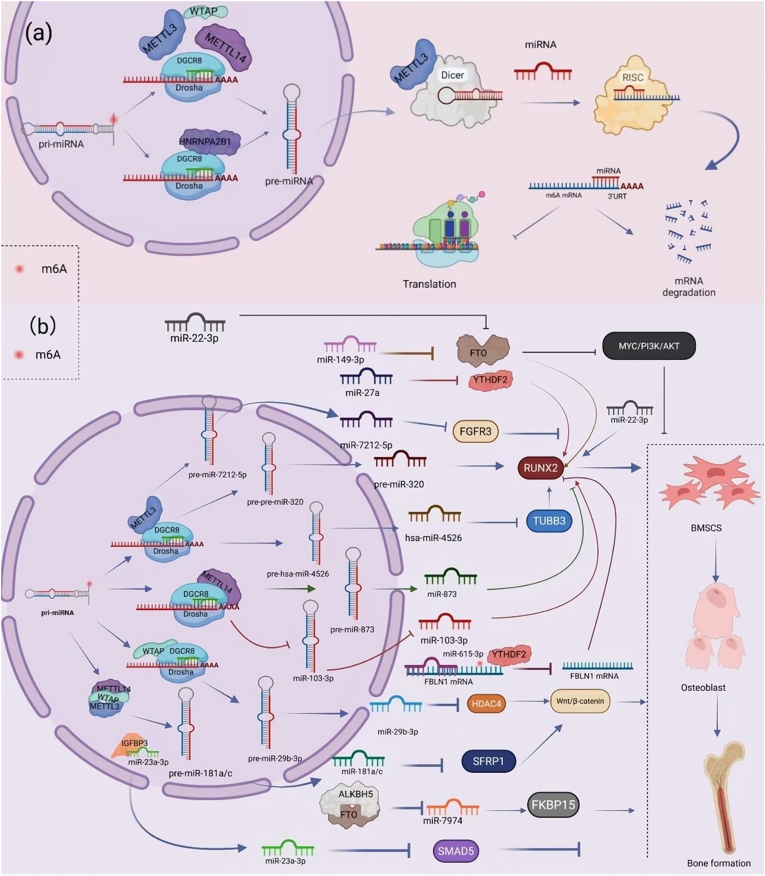
**Regulation of osteogenic differentiation by m6A modification of miRNA.** (a) **Canonical pathway of miRNA biogenesis.** In the nucleus, primary miRNAs (pri-miRNAs) are cleaved by the Drosha–DGCR8 microprocessor complex to form precursor miRNAs (pre-miRNAs), which are exported to the cytoplasm and further processed by Dicer into mature miRNAs. Mature miRNAs bind AGO2 to form the RNA-induced silencing complex (RISC), which targets complementary sequences in the 3′-UTR of mRNAs to repress translation or induce degradation. m6A methyltransferases such as METTL3, METTL14, WTAP, and HNRNPA2B1 facilitate pri-miRNA recognition and processing by DGCR8, while METTL3 also promotes Dicer-mediated pre-miRNA cleavage to accelerate miRNA maturation. (b) **Regulatory roles of miRNAs and m6A modifiers in osteogenic differentiation.** RUNX2 acts as the master regulator of osteogenesis by activating bone-related genes (e.g., osteocalcin, osteopontin). METTL3 promotes miR-4526 maturation to suppress TUBB3 and enhance RUNX2 expression, and relieves miR-320–mediated inhibition of RUNX2 by blocking its DGCR8-dependent processing. METTL14 exerts bidirectional regulation: it enhances miR-873 maturation to inhibit RUNX2, but also suppresses miR-103-3p maturation to promote it. miR-615-3p associates with YTHDF2 to destabilize FBLN1 mRNA and inhibit RUNX2, whereas miR-27a targets YTHDF2 to activate RUNX2. Similarly, miR-149-3p enhances RUNX2 by inhibiting FTO, and miR-22-3p directly upregulates RUNX2. The Wnt/β-catenin pathway is another key osteogenic axis. WTAP, together with METTL3/METTL14, catalyzes maturation of miR-181a/c, which represses SFRP1 to activate Wnt/β-catenin signaling. WTAP also promotes miR-29b-3p maturation, which inhibits HDAC4 and further enhances Wnt/β-catenin activity. Additional mechanisms include miR-22-3p promoting osteogenesis via FTO inhibition and MYC/PI3K/AKT blockade; miR-7212-5p and miR-23a-3p inhibiting osteogenesis by targeting FGFR3 and SMAD5 respectively; and FTO or ALKBH5 enhancing osteogenesis by repressing miR-7974 to increase FKBP15 expression.

The enrichment of m6A modifications within 3′-UTRs, which are regions densely populated by miRNA binding sites, suggests a close functional relationship between these two post-transcriptional regulators [34]. Beyond spatial colocalization, m6A exerts direct influence on miRNA biogenesis, including their processing, splicing, and maturation [35]. According to the canonical pathway described by Han et al., pri-miRNAs are cleaved in the nucleus by the Drosha–DGCR8 microprocessor complex into precursor miRNAs (pre-miRNAs). These are then exported to the cytoplasm, where Dicer further processes them into mature double-stranded miRNAs [36]. Mature miRNAs associate with AGO2 to form the RNA-induced silencing complex (RISC), which subsequently recognizes complementary 3′-UTR regions in target transcripts, inducing mRNA degradation or translation repression [37]. Several reports have also demonstrated that m6A modifications catalyzed by METTL3 facilitate miRNA maturation. METTL3-mediated methylation of pri-miRNAs enhances DGCR8 binding affinity, thereby accelerating the conversion of pri-miRNAs to pre-miRNAs [38]. In addition, Wang et al. reported that METTL3 promotes Dicer-dependent cleavage of pre-miRNAs, further expediting the generation of mature miRNAs [39]. Similar effects have been documented for other m6A writer proteins, including METTL14 [40] and WTAP (Fig. 2a) [41].

Although these studies establish a general framework, the precise impact of m6A on osteogenic differentiation is context-dependent and often divergent. For instance, Mi et al. demonstrated that METTL3 promotes m6A modification of pri-miR-7212-5p, enhancing its DGCR8-mediated processing into mature miR-7212-5p. This miRNA suppresses FGFR3 expression, ultimately impairing osteoblast differentiation and mineralization in vitro and delaying fracture healing in vivo [42]. In contrast, Song et al. reported that METTL3 enhances m6A modification of pri-miR-4526, facilitating its processing into miR-4526, which downregulates TUBB3, a negative regulator of osteogenesis. Upregulation of miR-4526 or silencing of TUBB3 increased RUNX2 and ALP expression, promoted mineralized nodule formation, and significantly improved osteogenic differentiation [43]. Xiao et al. used both in vivo and in vitro experiments to show that METTL3, as an m6A methyltransferase, interacts with DGCR8 to promote the m6A-dependent maturation of pri-miR-324-5p. The mature miR-324-5p, by targeting and inhibiting ELAVL1, facilitates osteogenic differentiation while the differentiation of osteoclasts and adipocytes [44]. Interestingly, METTL3 can also function in the opposite direction. Yan et al. found that METTL3-mediated m6A methylation of pre-miR-320 prevents its maturation, thereby relieving the miR-320-mediated inhibition of RUNX2. This, along with direct stabilization of RUNX2 mRNA via m6A methylation, promotes osteoblast differentiation and bone formation [45]. In addition, METTL3 mediates the direct m6A methylation of the RUNX2 mRNA, enhancing its stability and expression, thereby facilitating osteoblastic differentiation and maturation.

The multifarious and sometimes opposing functions of METTL3 underscore the complexity of m6A-miRNA interactions in osteogenesis. Similarly, METTL14 acts in a context-specific manner. Sun et al. showed that miR-103-3p inhibits osteoblast activity by targeting METTL14, whereas METTL14-dependent m6A modification limits DGCR8-mediated processing of miR-103-3p, ultimately favoring osteogenesis [46]. Conversely, Dong et al. reported that METTL14 binds DGCR8 and catalyzes m6A modification of pri-miR-873, facilitating its maturation. Elevated miR-873 levels suppressed bone marrow mesenchymal stem cell (BMSC) proliferation and differentiation, as evidenced by reduced S-phase cell populations and downregulation of osteogenic markers. Silencing METTL14 alleviated this repression, restoring BMSC proliferation and osteogenic potential [47].

These studies suggest that METTL3 and METTL14 influence osteogenic differentiation by either facilitating or blocking miRNA maturation. WTAP, an essential partner in the m6A methyltransferase complex, also contributes to this regulatory process by guiding m6A deposition and modulating miRNA biogenesis. For example, You et al. [48] reported that WTAP associates with METTL3 and METTL14 to form a methyltransferase complex that modifies pri-miR-181a and pri-miR-181c. These modifications accelerate their processing into mature miR-181a/c, which in turn activate the Wnt/β-catenin signaling pathway through direct inhibition of the 3′-UTR of SFRP1 (a Wnt antagonist), thereby enhancing osteogenic differentiation. Similarly, Liu et al. [41] found that WTAP interacts with DGCR8 to promote the m6A modification of pri-miR-29b-3p. This modification facilitates its maturation into miR-29b-3p, which suppresses HDAC4 expression at the 3′-UTR level. By relieving HDAC4-mediated repression, miR-29b-3p further activates the Wnt/β-catenin pathway, driving osteogenic differentiation.

While these findings highlight the importance of methyltransferases, the role of m6A reader proteins in this context has also become increasingly clear through their ability to detect modifications and facilitate functional decoding. Yang et al. [49] uncovered a mechanism in Wharton's jelly-derived mesenchymal stem cells (WJMSCs) whereby FBLN1 mRNA modulates osteogenic differentiation and bone regeneration. They found that the 3′-UTR of FBLN1 harbors both m6A and miR-615-3p binding sites. While mutations eliminating the m6A sites enhanced FBLN1 transcript stability and promoted osteogenesis, the reader protein YTHDF2 was found to recognize the m6A sites and to cooperate with miR-615-3p to destabilize the FBLN1 mRNA, limiting its expression and ultimately inhibiting osteogenic differentiation. Consistently, Yuan et al. [50] reported that miR-27a directly targets YTHDF2 to suppress its expression and activity, leading to a global reduction in m6A methylation levels, thereby favoring BMSC osteogenesis while restraining adipogenesis. Additional evidence was provided by Lai et al. [51], who demonstrated that IGFBP3 exerts an inhibitory effect on osteoblast differentiation both in vitro and in vivo. Mechanistically, IGFBP3-mediated m6A modification stabilized and increased the abundance of miR-23a-3p, which repressed SMAD5 and subsequently attenuated osteogenic differentiation and fracture repair.

Collectively, these findings suggest that both methyltransferases and readers shape the landscape of miRNA regulation via m6A, thereby influencing osteogenic fate decisions and osteoporosis progression. However, demethylases such as FTO and ALKBH5 also participate, adding a reversible and dynamic dimension to this regulatory system. Li et al. [52] observed that miR-149-3p is highly expressed during osteoblast differentiation but decreases during adipogenic commitment. By repressing its downstream target FTO, miR-149-3p skews BMSC differentiation toward osteoblasts while limiting adipocyte formation, a mechanism further validated in osteoporosis models. Similarly, Zhang et al. [53] demonstrated that miR-22-3p enhances osteogenic differentiation by increasing the expression of RUNX2, osteocalcin, and osteopontin in BMSCs. Notably, BMSC-derived extracellular vesicles (EVs) transport miR-22-3p, which targets and suppresses FTO, thereby blocking the MYC/PI3K/AKT pathway and promoting osteoblastogenesis. In another study, Zheng et al. [54] reported that forced overexpression of FTO or ALKBH5 decreased the stability of miR-7974, leading to upregulation of its downstream target FKBP15. Elevated FKBP15 levels impaired the osteogenic differentiation of dental follicle stem cells (DFSCs). Conversely, silencing FKBP15 or enhancing miR-7974 expression restored calcium deposition and increased the expression of osteogenic markers such as osteocalcin (OCN) and osteopontin (OPN), thereby improving osteogenesis (Fig. 2b).

In summary, m6A modification can shape miRNA maturation and function in a dynamic manner through the coordinated activity of methyltransferases, reader proteins, and demethylases, ultimately influencing osteogenic differentiation via signaling cascades and target gene regulation. Importantly, this process is not strictly unidirectional, as the same enzyme (e.g., METTL3 or METTL14) can exert opposite effects depending on the miRNA involved, the specific signaling pathway, or the physiological versus pathological context. This bidirectionality underscores the complexity of epitranscriptomic regulation and provides a framework for exploring novel therapeutic strategies for metabolic bone disorders. However, in light of the complex and sometimes contradictory outcomes that have been described to date, further mechanistic studies are required to fully unravel how m6A-dependent miRNA regulation governs osteogenic differentiation.

### 3.3 The role of m6A-Modified lncRNAs in osteogenic differentiation

High-throughput sequencing studies reveal that lncRNAs harbor numerous m6A peaks: approximately 225–2627 in human cell lines and 101–1243 in mouse cell lines, with distribution patterns showing clear transcript-specific enrichment [55]. These findings highlight the widespread involvement of m6A modifications in lncRNA biology. In recent years, particular attention has been directed to METTL3-mediated regulation of lncRNAs in the context of osteogenic differentiation. First, lncRNAs function as competitive endogenous RNAs (ceRNAs), sequestering miRNAs and thereby relieving repression on downstream mRNA targets [56]. For instance, LINC00657, which is ubiquitously expressed and contributes to chromosome stability, is stabilized and upregulated by METTL3-mediated m6A modification. Acting as a ceRNA, LINC00657 can sequester miR-144-3p, abrogating its repression of BMPR1B and ultimately promoting osteogenic differentiation [57]. Similarly, the osteoporosis-associated lncRNA MIR99AHG undergoes METTL3-dependent methylation, which diminishes its ability to serve as a molecular sponge for miR-4660, leading to miR-4660 overexpression. This upregulates RUNX2, a master osteogenic transcription factor, thereby facilitating BMSC differentiation [58]. In periodontal ligament stem cells (pPDLSCs) derived from periodontitis patients, METTL3-mediated methylation enhances the stability of lncRNA CUTALP, which competes with miR-30b-3p to derepress Runx2, again promoting osteogenesis [59]. Another example is lncRNA XIST, which suppresses miR-302a-3p. Since miR-302a-3p inhibits USP8, METTL3-dependent methylation of lncRNA XIST enhances this lncRNA–miRNA interaction, lowering miR-302a-3p levels and sustaining osteogenic differentiation. Overexpression of miR-302a-3p blocks osteogenesis, whereas restoring USP8 expression reverses this effect [60].

**Fig. 3:**
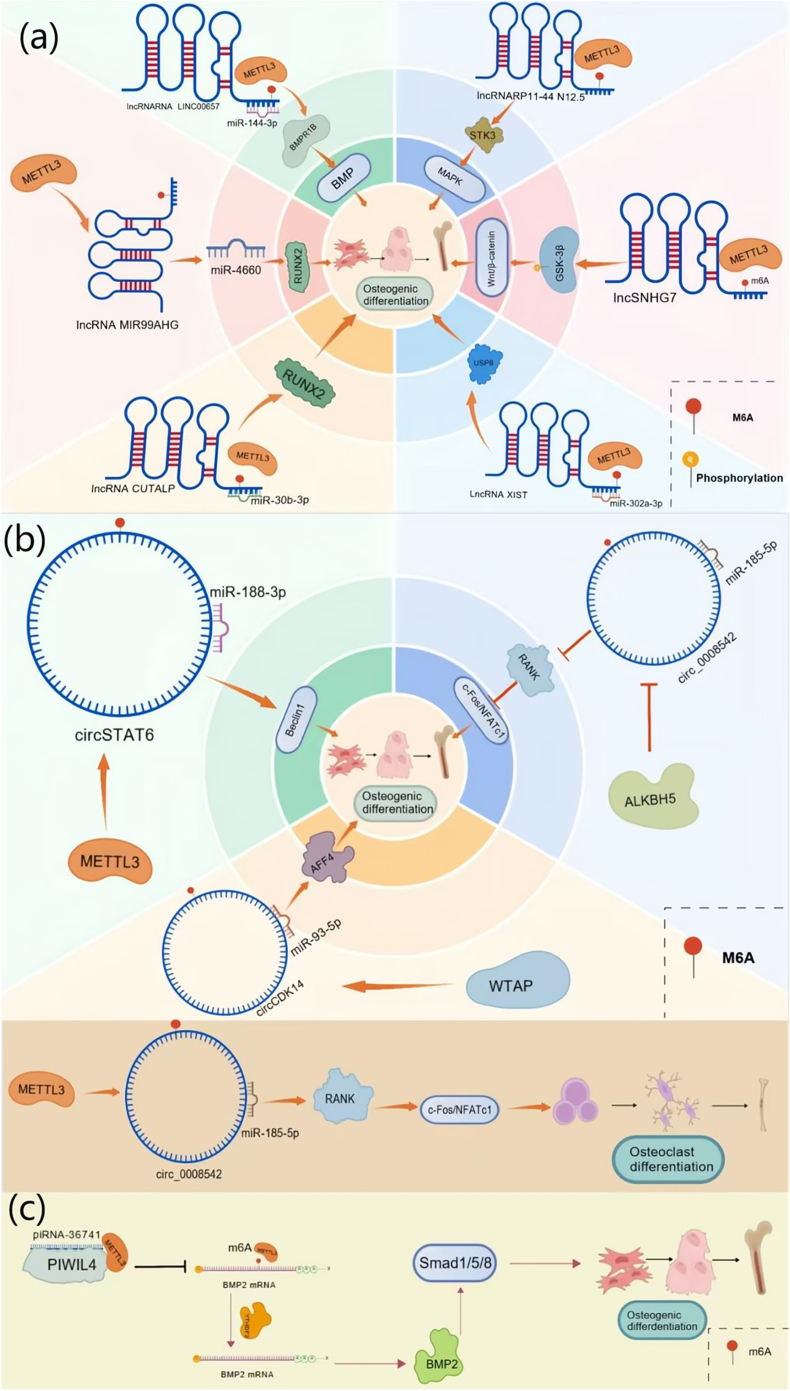
**The role of lncRNA, circRNA, and piRNA m6A modification in osteogenic differentiation. (a) m6A-modified lncRNAs in osteogenic differentiation.** lncRNAs act as competing endogenous RNAs (ceRNAs) to sequester miRNAs and derepress osteogenic genes. LINC00657 sponges miR-144-3p to upregulate BMPR1B and promote osteogenesis. METTL3-mediated m6A modification of MIR99AHG reduces its miR-4660 sponging, increasing miR-4660 levels to activate RUNX2. METTL3 also stabilizes CUTALP, enabling it to compete with miR-30b-3p and enhance RUNX2 expression. Similarly, m6A-modified XIST suppresses miR-302a-3p and elevates USP8, facilitating osteogenic differentiation. Beyond miRNA sponging, m6A marks modulate lncRNA–protein interactions: METTL3-methylated SNHG7 activates Wnt/β-catenin signaling via GSK-3β phosphorylation in hDPSCs, while METTL3-stabilized RP11-44N12.5 regulates STK3 to activate the MAPK pathway and promote osteogenesis in hASCs. (b) **m6A-modified circRNAs in osteogenic differentiation.** circRNAs also function as miRNA sponges under m6A regulation. circSTAT6 sequesters miR-188-3p to enhance Beclin1 expression and osteogenesis. WTAP-mediated m6A methylation stabilizes circCDK14, which binds miR-93-5p and elevates AFF4, promoting osteogenic differentiation. Conversely, m6A demethylation by ALKBH5 weakens circ0008542–miR-185-5p binding, suppressing RANK/c-Fos/NFATc1–driven osteoclastogenesis and favoring osteogenesis. In contrast, METTL3-mediated methylation of circ0008542 reduces miR-185-5p expression, derepressing RANK and activating osteoclastogenic signaling. (c) **m6A-modified piRNAs in osteoporosis regulation.** piRNA-36741 forms a complex with PIWIL4 that competes with METTL3, reducing m6A methylation of BMP2 mRNA. This prevents YTHDF2-mediated degradation, stabilizes BMP2 transcripts, activates Smad1/5/8 signaling, and promotes osteogenic differentiation of BMSCs.

In addition to ceRNA mechanisms, m6A modifications can regulate protein binding to lncRNAs, influencing downstream signaling cascades [61]. For example, lncSNHG7, when modified by METTL3, promotes osteogenic and odontogenic differentiation of human dental pulp stem cells (hDPSCs) via activation of the Wnt/β-catenin pathway, partly through phosphorylation of GSK-3β [62]. Similarly, RP11-44N12.5 becomes stabilized by METTL3-catalyzed m6A modification, enabling it to regulate STK3 and activate the MAPK pathway, thereby driving osteogenic differentiation of human adipose-derived stem cells (hASCs) (Fig. 3a) [63].

These publications primarily emphasize the role of METTL3 as a major regulator of lncRNA m6A methylation in the context of osteogenesis. In contrast, the contributions of other methyltransferases, demethylases, and m6A readers remain insufficiently explored. Addressing these gaps in knowledge will be essential to fully delineate the complexity of lncRNA-m6A-osteogenesis regulatory networks.

### 3.4 Research progress on the m6A modification of circRNAs in osteoporosis

In addition to miRNAs and lncRNAs, circRNAs are also subject to m6A modification and participate in bone metabolism. CircRNAs are increasingly recognized as multifunctional regulators of gene expression. Their primary modes of action include: (i) functioning as miRNA sponges, sequestering specific miRNAs and preventing them from repressing target mRNAs; and (ii) directly interacting with proteins, thereby modulating protein localization, stability, or protein–protein interactions [64]. Luo et al. [65] demonstrated that circSTAT6 promotes osteogenic differentiation of BMSCs by serving as a sponge that sequesters miR-188-3p, leading to the upregulation of Beclin1, a crucial mediator of osteogenesis under miR-188-3p control. Importantly, METTL3 stabilizes circSTAT6 transcripts via m6A modification, further amplifying this pro-osteogenic effect. Similarly, Chen et al. [66] reported that METTL3 can interact with nucleolar protein 2 (NOP2) and eukaryotic translation initiation factor 3 subunit A (EIF3A) to indirectly enhance m6A deposition on circCTTN. Elevated expression and methylation of circCTTN, induced by METTL3 overexpression, promoted osteogenic differentiation in human umbilical cord mesenchymal stem cells (hUCMSCs). While the specific downstream mechanism of circCTTN remains to be elucidated, these findings underscore the importance of METTL3-mediated m6A regulation as a determinant of circRNA functionality. Additional evidence comes from Zhao et al. [67], who found that WTAP promotes the stability of circCDK14 by increasing its m6A methylation and facilitating its interaction with the reader protein IGF2BP3. Stabilized circCDK14 in turn acts as a sponge for miR-93-5p, thereby enhancing the expression of AFF4, a transcriptional regulator implicated in osteogenesis. Since miR-93-5p normally suppresses AFF4 by binding to its 3′-UTR, silencing AFF4 markedly reduced the osteogenic potential of ligamentum flavum cells. Notably, the influence of m6A-modified circRNAs is not restricted to the context of osteogenesis, as it also influences osteoclast differentiation [68]. For example, METTL3 has been shown to methylate circ0008542 at nucleotide position 1956, increasing its affinity for miR-185-5p. By sequestering miR-185-5p, circ0008542 can relieve the repression of its downstream target RANK, thereby activating osteoclastogenic signaling pathways (e.g., c-Fos/NFATc1) and promoting bone resorption. In contrast, the demethylase ALKBH5 can reverse this process by removing the m6A methylation mark, reducing circ0008542 binding to miR-185-5p, suppressing osteoclast activation, and favoring osteogenesis (Fig. 3b).

Collectively, these studies highlight circRNAs as key epitranscriptomic regulators of bone metabolic homeostasis. However, further research is needed to clarify their roles in osteoporosis and the effects of m6A modification on these dynamics.

### 3.5 Preliminary research on the m6A modification of piRNAs in osteoporosis

piRNAs are a distinct class of small non-coding RNAs, typically 26–31 nucleotides in length, that exert gene regulatory functions through interaction with PIWI-like (PIWIL) proteins. Although their role in somatic tissues is less understood compared to the germline, emerging evidence suggests piRNAs may also influence skeletal biology. A recent study [69] provided the first evidence linking piRNA m6A regulation to bone metabolism. The authors reported that piRNA-36741 binds to PIWIL4, forming a complex that competes with METTL3 for substrate interaction. By blocking METTL3 activity, this complex reduces m6A methylation of BMP2 mRNA, thereby preventing its recognition and degradation by the reader protein YTHDF2. As a result, BMP2 mRNA stability and expression are increased, activating the Smad1/5/8 signaling pathway, upregulating osteogenesis-related genes, and enhancing BMSC differentiation into osteoblasts. Functionally, this mechanism alleviated osteoporosis phenotypes in experimental models (Fig. 3c).

At present, this is the only published report examining piRNA-mediated modulation of m6A in the context of osteoporosis. The proposed mechanism through which piRNAs interact with PIWI proteins to antagonize METTL3, stabilize BMP2 transcripts, and promote osteogenesis remains intriguing but requires independent validation. Given the limited scope of current data, comprehensive studies using larger models and multi-omics approaches are urgently needed to clarify the broader regulatory roles of piRNAs in skeletal homeostasis and osteoporosis progression.

In summary, the most in-depth and systematic current research on ncRNAs in osteoporosis and osteogenic differentiation has addressed the functions of miRNAs. In contrast, studies on lncRNAs and circRNAs have focused primarily on their interactions with methyltransferases. Further exploration of the roles of demethylases and reader proteins is needed. Additionally, the regulatory effects of lncRNAs and circRNAs in osteogenic differentiation often involve their functioning as “miRNA sponges,” sequestering and attenuating miRNA-mediated suppression of target genes and thereby indirectly promoting the expression of osteogenic-related genes and the osteogenic differentiation process. This indicates that, compared to other non-coding RNA types, miRNAs occupy a more central position in the regulatory network controlling osteogenic differentiation.

## 4 Effects of histone modifications on osteoporosis mediated by ncRNAs

Currently, there is limited research on RNA modifications other than m6A in osteoporosis. Studies have shown that modifications such as 5-methylcytosine (m^5^C), pseudouridylation (Ψ), and N^1^-methyladenosine (m^1^A) play significant roles in the regulation of various physiological and pathological processes. However, their specific functions in the development and progression of osteoporosis require further investigation. Meanwhile, histone modifications, which regulate osteogenic differentiation by influencing the expression and function of RNAs, are becoming a research hotspot. There are various forms of histone modifications, including methylation, acetylation, phosphorylation, and ubiquitination. Among these, histone acetylation and deacetylation have received increasing attention in the context of the regulation of bone metabolism. Histone acetylation can induce gene activation by increasing the accessibility of DNA to the transcriptional machinery [70] In contrast, histone deacetylation results in chromatin condensation, associated with gene inactivation, thereby affecting the expression of osteogenesis-related genes and the bone formation process [71].

Histone acetylation is a key epigenetic mechanism that regulates osteogenesis and bone homeostasis. [72,73]. Of particular interest is the influence of histone deacetylation on the occurrence and progression of osteoporosis through the regulation of ncRNAs. Studies have shown that inhibitors of HDAC can induce hyperacetylation of histones in skeletal myoblasts, leading to increased secretion of extracellular vesicles (EVs) enriched with miR-873-3p. Once internalized by BMSCs, miR-873-3p promotes osteogenic differentiation and reduces bone loss by suppressing CNN2 expression [74]. Mechanical unloading, a major contributor to osteoporosis, is also associated with acetylation modifications. Under unloading conditions, downregulation of HDAC6 increases acetylation of the miR-375-3p promoter and enhances its transcription. The upregulated targets of miR-375-3p inhibit LRP5, thereby reducing Wnt/β-catenin signaling, impairing bone microvascular structure, and suppressing osteogenesis [75]. In contrast, it has been found that unloading can also upregulate overall HDAC levels, inhibit acetylation of the JAG1 promoter, and silence the JAG1-NOTCH pathway, thereby suppressing the proliferation and osteogenic differentiation of BMSCs and promoting bone loss [76]. In addition, non-coding RNAs can act synergistically with epigenetic modifications to regulate bone homeostasis. For example, miR-29a in osteoblasts can simultaneously suppress RANKL expression to reduce osteoclast signaling, inhibit PCAF activity, and lower the levels of H3K27ac in the CXCL12 promoter, thereby reducing osteoclast chemotaxis and activation [77].

In conclusion, histone acetylation and deacetylation coordinately regulate osteogenesis and bone homeostasis. Acetylation promotes osteogenic differentiation by increasing H3K9ac in the promoter regions of key genes (RUNX2, BMPs, Wnts), resulting in activation of BMP/Smad and Wnt signaling. Deacetylation primarily modulates ncRNAs, thereby influencing osteogenic and osteoclastic processes. This interplay between histone modification and ncRNAs underscores the broader epigenetic landscape controlling bone metabolism. Although, to date, m6A has been the most extensively studied form of RNA modification involved in the regulation of ncRNAs during osteogenesis, there is a need for further exploration of the synergistic relationships among other epitranscriptomic or histone modifications and m6A, with the formation of a multi-layered regulatory network affecting bone metabolism.

## 5 Discussion

m6A modifications are both reversible and bidirectionally regulated. It is thus important, in the research or treatment of osteoporosis, not to blindly reduce the overall m6A modification levels or directly inhibit the activity of a specific m6A-related enzyme solely based on abnormal expression of a particular ncRNA. Such nonspecific interventions may trigger widespread dysregulation of gene expression, with unpredictable biological effects. Therefore, a more rational strategy should involve targeted regulation of the status of m6A modification or expression levels based on the functional characteristics of specific ncRNAs, enabling precse intervention in osteoporosis development. Moreover, different ncRNAs differ markedly in their stability, processing efficiency, and downstream regulatory mechanisms after m6A modification. This also poses significant challenges for the clinical translation and targeted therapeutic application of m6A modification in osteoporosis. To address this issue, an “axis-associated regulation” model was proposed, integrating m6A modifications, ncRNAs, and downstream target genes to elucidate the underlying mechanisms. Analysis of the interactions among the components of this axis, together with the implementation of specific directional regulation, may lead to the development of more precise and efficient strategies for treating osteoporosis. Current evidence indicates the centrality of the Wnt/β-catenin and BMP/Smad pathways in osteogenic differentiation. The activity of these pathways is closely linked to the degree of ncRNA m6A modification. For example, the three axes mentioned earlier, namely, WTAP-miR-181a/c-SFRP1, WTAP-miR-29b-3p-HDAC4, and METTL3-lncSNHG7-GSK-3β", can collectively activate Wnt signaling. Among these, the levels of mature miR-181a/c and miR-29b-3p in the peripheral blood and the m6A modification status of lncSNHG7 may serve as potential indicators of Wnt pathway activity. Additionally, the piRNA-36741-BMP2-Smad1/5/8 and METTL3-LINC00657-BMPR1B axes are critical to the regulation of the BMP signaling pathway. The expression level of piRNA-36741 and the degree of m6A modification of LINC00657 have potential as biomarkers for assessing osteogenic function.

These findings suggest that it would be possible to identify factors associated with osteoporosis pathogenesis by measuring the expression levels of ncRNAs associated with these pathways. Significant reductions in the levels of ncRNAs related to the Wnt/β-catenin or BMP/Smad pathways would suggest the possibility of mitigating osteoposisis development or progression by inhibiting these pathways. Conversely, elevated levels of these ncRNAs could preliminarily exclude the pathways as being fundamentally involved in disease pathogenesis, thereby avoiding the likelihood of misdiagnosis and overtreatment. In terms of circRNAs, their closed-loop structures confer greater in vivo stability, while these ncRNAs tend to be more tissue-specific. Therefore, the levels of circRNA may have potential as indicators for the early diagnosis of osteoporosis. Compared to traditional bone density testing, this “signaling pathway biomarker axis"-based detection method represents a direct reflection of changes at the molecular level, enabling greater sensitivity and more effective predictive value.

In summary, systematic research on the “m6A modification-ncRNA-downstream target gene” axis may contribute to the establishment of a framework for precision diagnosis and treatment of osteoporosis. First, due to the bidirectional and reversible regulation of m6A modifications and variations in the effects of m6A modification on ncRNAs under different conditions, it is clear that regulation is both multi-level and synergistic. Additionally, this axis-based regulation may offer advantages in terms of detectability and therapeutic monitoring. However, challenges remain in terms of clinical translation, including a lack of efficient techniques for the detection of ncRNA and m6A levels in peripheral blood, the susceptibility of ncRNAs to degradation during in vivo delivery, and the need for safety assessments to determine whether the activation of m6A-related enzymes or pathways affects other organs in the body.

## 6 Summary and outlook

In summary, accumulating evidence indicates that m6A modification and ncRNAs act in concert to regulate bone metabolism and the pathogenesis of osteoporosis. The presence of specific regulatory axes, such as miR-29b-3p/HDAC4 and circ0008542/miR-185-5p/RANK, illustrates the ability of m6A-dependent ncRNA networks to fine-tune osteogenic and osteoclastic differentiation. These interactions comprise an interconnected “m6A-ncRNA-target gene” regulatory framework that shapes the epigenetic landscape of bone remodeling (see Table 1).

**Table 1:** Regulation of osteoporosis by m6A modification through ncRNA.

M6A	RNA	Biological function	Mechanism	References
METTL3	miR-7212-5p	Inhibit osteogenesis	METTL3↑miR-7212-5p↑	[42]
METTL3	hsa-miR-4526	Promote osteogenesis	METTL3↑hsa-miR-4526↑TUBB3↓RUNX2↑	[43]
METTL3	miR-324-5p	Promote osteogenesis	METTL3↑miR-324-5p↑ELAVL1↓	[44]
METTL3	miR-320	Inhibit osteogenesis	METTL3↑miR-320↓RUNX2↑	[45]
METTL14	miR-103-3p	Inhibit osteogenesis	METTL14↑miR-103-3↓RUNX2↑	[46]
METTL14	miR-873	Inhibit osteogenesis	METTL14↑miR-873↑	[47]
WTAP	miR-181a/c	Promote osteogenesis	WTAP↑miR-181a/c↓Wnt/β-catenin↑	[48]
WTAP	miR-29b-3p	Promote osteogenesis	WTAP↑miR-29b-3p↓Wnt/β-catenin↑	[41]
YTHDF2	miR-615-3p	Inhibit osteogenesis	YTHDF2↑miR-615-3p↓	[49]
YTHDF2	miR-27a	Promote osteogenesis	miR-27a↑YTHDF2↓	[50]
IGFBP3	miR-23a-3p	Inhibit osteogenesis	IGFBP3↑miR-23a-3p↑SMAD5↓	[51]
FTO	miR-149-3p	Promote osteogenesis	miR-149-3p↑FTO↓	[52]
FTO	miR-22-3p	Promote osteogenesis	miR-22-3p↑FTO↓RUNX2↑	[53]
FTO/ALKBH5	miR-7974	Inhibit osteogenesis	FTO/ALKBH5↑miR-7974↓FKBP15↑	[54]
METTL3	LINC00657	Promote osteogenesis	METTL3↑LINC00657↑miR-144-3p↓BMP↑	[57]
METTL3	MIR99AHG	Promote osteogenesis	METTL3↑MIR99AHG↓miR-4660↑RUNX2↑	[58]
METTL3	lncRNA CUTALP	Promote osteogenesis	METTL3↑lncRNA CUTALP↑miR-30b-3p↓RUNX2↑	[59]
METTL3	lncRNAXIST	Promote osteogenesis	METTL3↑lncRNAXIST↑miR-302a-3p↓USP8↑	[60]
METTL3	lncSNHG7	Promote osteogenesis	METTL3↑lncSNHG↑Wnt/β-catenin↑	[62]
METTL3	lncRNARP11-44N12.5	Promote osteogenesis	METTL3↑lncRNARP11-44N12.5↑STK3↑MAPK↑	[63]
METTL3	circSTAT6	Promote osteogenesis	METTL3↑circSTAT6↑miR-188-3p↓Beclin1↑	[65]
METTL3	circCTTN	Promote osteogenesis	METTL3↑circCTTN↑RUNX2↑	[66]
WTAP	circCDK14	Promote osteogenesis	METTL3↑circCDK14↑miR-93-5p↓AFF4↑	[67]
METTL3	circ0008542	Promote osteoclast differentiation	METTL3↑circ_0008542↑miR-185-5p↓RANK↑	[68]
ALKBH5	circ0008542	Promote osteogenesis	ALKBH5↑circ_0008542↓miR-185-5↑RANK↓	[68]
METTL3	piRNA36741	Promote osteogenesis	METTL3↑piRNA-36741↓BMP2↑Smad1/5/8↑	[69]

Despite these advances, several gaps remain in the extant literature. Current research is still dominated by studies of m6A writers (e.g., METTL3, METTL14), while the roles of erasers (FTO, ALKBH5) and readers (YTH family proteins, IGF2BPs) are less well defined. Investigations into circRNA- and piRNA-mediated m6A regulation are still in their infancy, with relatively few experimental reports available at present. Moreover, the signaling networks influenced by m6A–ncRNA interactions are highly complex, requiring integrative systems-level analyses to map their hierarchical structure. Although preliminary findings suggest that m6A–ncRNA signatures hold promise as diagnostic biomarkers or therapeutic targets for osteoporosis, translation into clinical practice remains in its infancy. Intervention strategies targeting core regulators such as METTL3/METTL14 have yet to be systematically developed.

Future studies should aim to systematically dissect the interplay between m6A modification and non-coding RNAs. In particular, building comprehensive regulatory network models that integrate the activities of m6A-associated enzymes, including the methyltransferases METTL3 and METTL14, the demethylases FTO and ALKBH5, as well as recognition proteins such as the YTH family, will be essential to clarify their layered roles in osteogenic regulation. The functions of circRNAs and piRNAs under m6A control remain only superficially understood, with most findings still at an exploratory stage; therefore, detailed mechanistic investigations are urgently needed to delineate their contributions to osteoporosis pathophysiology. Importantly, research on translation should focus on the development of precision medicine that includes the evaluation of m6A-modified ncRNAs in peripheral blood or extracellular vesicles as early diagnostic markers and therapeutic targets. Combining these molecular axes with the Wnt/β-catenin and BMP/Smad pathways may enable the establishment of signal-axis-based molecular subtyping of osteoporosis, thus offering a more accurate and individualized framework for both diagnosis and treatment.

## CRediT authorship contribution statement

**Yongbin Wang:** Writing – review & editing, Writing – original draft, Visualization, Supervision, Software, Resources, Methodology, Investigation, Formal analysis, Data curation, Conceptualization. **Baicheng Ma:** Writing – review & editing, Writing – original draft, Supervision, Funding acquisition, Conceptualization. **Jiashuo Qiu:** Writing – original draft. **Likun Yu:** Validation, Software. **Jianjun Xiong:** Writing – original draft, Supervision. **Qianfu Yu:** Writing – original draft, Validation. **Xingnuan Li:** Writing – original draft, Software. **Haichun Liao:** Writing – original draft, Conceptualization. **Youen Huang:** Writing – review & editing, Writing – original draft. **Shan He:** Writing – review & editing, Writing – original draft, Visualization, Supervision, Funding acquisition, Data curation, Conceptualization.

## Data availability statement

The data that support the findings of this study are available from the corresponding author upon reasonable request.

## Funding

Our research was funded by the 10.13039/501100001809National Natural Science Foundation of China (No. 82360443 awarded to Baicheng Ma), Science and Technology Program of Jiangxi Provincial Administration of Traditional Chinese Medicine (No. 2022B922 awarded to Shan He) and Jiangxi Provincial Natural Science Foundation (No. 20232BAB206051 awarded to Baicheng Ma), Jiangxi Provincial Natural Science Foundation (No. 20242BAB25457 awarded to Xingnuan Li), key research and development project of Jiujiang City, Jiangxi Province (No. S2024ZDYFN0009 awarded to Xingnuan Li).

## Declaration of competing interest

The authors declare that they have no known competing financial interests or personal relationships that could have appeared to influence the work reported in this paper.
